# Micronutrient supplementation among adults following restrictive diets in Riyadh: a cross-sectional study

**DOI:** 10.3389/fnut.2026.1686609

**Published:** 2026-02-12

**Authors:** Rehab Aldahash, Haifa Alturki, Joury Dabbour, Shahad Aldossari, Shahad Alonazi, Sarah Aldhfayan

**Affiliations:** Department of Health Sciences, College of Health and Rehabilitation Sciences, Princess Nourah Bint Abdulrahman University, Riyadh, Saudi Arabia

**Keywords:** dietary regimens, iron, micronutrients, Saudi Arabia, supplementation, vitamin D

## Abstract

**Background:**

The growing adoption of restrictive dietary regimens has raised concerns regarding micronutrient adequacy; however, evidence from Saudi Arabia remains limited.

**Methods:**

A cross-sectional study was conducted among 400 Saudi adults aged 18–45 years living in Riyadh between December 2024 and March 2025. Participants completed a validated online questionnaire capturing sociodemographic characteristics, dietary regimen, and micronutrient supplementation patterns. Statistical analyses comprised descriptive statistics, the chi-squared test, and Fisher’s exact tests to assess associations between dietary regimens and supplement use, and a multivariable logistic regression analysis to evaluate the association between diet adherence duration and micronutrient consumption.

**Results:**

Overall, 45.1% of participants reported using micronutrient supplements. Vitamin D was the most frequently consumed vitamin, while iron was the most commonly used mineral across all diet types. Supplement use was highest among individuals following gluten-free (75%), ketogenic (66.7%), and low-carbohydrate (64.1%) diets. Significant associations were identified between specific dietary regimens and supplementation with vitamins A, D, K, and B12 and minerals, including calcium, potassium, and selenium. Additionally, longer adherence to certain dietary regimens, particularly intermittent fasting and the combined “Other Diets” group, was significantly associated with higher odds of micronutrient supplementation.

**Conclusion:**

Micronutrient supplementation is highly prevalent among individuals following restrictive diets in Riyadh, with specific regimens such as gluten-free, ketogenic, and low-carbohydrate diets showing greater dependence on supplements. Moreover, the increased likelihood of supplement use among individuals adhering to certain diets for longer durations highlights the significance of monitoring nutritional adequacy over time. These findings underscore the need for targeted dietary counselling and public health strategies that promote balanced nutrition according to popular diet trends.

## Introduction

The increasing adoption of diverse dietary regimens, whether for health, ethical, or personal reasons, has drawn global attention to their potential impact on nutritional status (1, 2). In Saudi Arabia, rapid urbanization and the influence of Western dietary habits have contributed to major shifts in eating patterns, including higher consumption of processed and fast foods alongside a growing interest in health-conscious diets (3). A recent study identified the most commonly followed dietary regimens in Saudi Arabia as the ketogenic diet, intermittent fasting, gluten-free diet, and calorie restriction, with vegetarianism also gaining popularity (4, 5).

While these diets may offer certain benefits, they also present nutritional challenges. The ketogenic diet, though sometimes effective for medical conditions, has shown inconsistent long-term weight loss outcomes and is often difficult to sustain due to its restrictive nature (6). Intermittent fasting and calorie restriction may require careful planning to ensure adequate nutrient intake (7, 8). Similarly, vegetarian and vegan diets, if not properly managed, can lead to deficiencies in key vitamins such as B12 and D (9).

Micronutrients are essential vitamins and minerals required in small quantities, yet they are fundamental to overall health and wellbeing (10). They are available in various forms, including tablets, capsules, and liquids, and may consist of a combination of vitamins, minerals, herbs, and other compounds (11). These nutrients support various physiological functions, including metabolism, immune response, and bone health (12). They are also directly associated with mental performance, emotional stability, and energy levels (11). In Saudi Arabia, the use of dietary supplements is widespread. A study conducted in Riyadh found that 63.2% of participants reported current or past use of nutritional supplements (13), with 53.6% of users being female and 42.5% male (14).

There is a well-established relationship between dietary regimen and vitamin intake, as different diets can influence both the quantity and type of vitamins consumed. For example, studies in Hong Kong and India have reported that up to 80% of vegans are deficient in vitamin B12 and iron, nutrients found naturally only in animal-based foods (15–17). Similarly, ketogenic diets may be associated with deficiencies in vitamin D, magnesium, calcium, iron, phosphorus, and potassium, due in part to reduced sun exposure and altered fat metabolism (18, 19). Despite growing global attention to the nutritional consequences of various dietary regimens, few studies have examined their micronutrient implications in the Saudi context. This lack of localized data on how specific dietary regimens influence micronutrient intake creates a gap in public health knowledge, particularly in populations experiencing rapid dietary transitions. In line with these gaps, the present study aimed to assess the prevalence of commonly practiced dietary regimens among adults in Riyadh, determine the extent of micronutrient supplementation within this population, and examine the association between dietary patterns and micronutrient intake. Accordingly, the study aimed to address the overarching research question: To what extent do the type and duration of dietary regimens influence micronutrient supplementation among adults in Riyadh?

## Materials and methods

### Study design and participants

A cross-sectional study was conducted among Saudi adults aged 18–45 years residing in Riyadh, Saudi Arabia, between December 2024 and March 2025. Participants were recruited using non-probability convenience sampling through online platforms (WhatsApp, X, and Telegram). Adults older than 45 years were excluded to minimize age-related variability in nutrient absorption and dietary behavior. Of the 412 questionnaires received, 400 valid responses were included in the final analysis. Participants who were pregnant or breastfeeding were excluded to avoid confounding due to altered nutritional requirements.

### Data collection tool

Data were collected using an online questionnaire that assessed sociodemographic characteristics, dietary regimen practices, and micronutrient supplementation. The instrument underwent content validation by five academic experts, and its internal consistency was confirmed (Cronbach’s *α* = 0.80). Although no pilot study was conducted, expert feedback was used to revise the questionnaire for clarity and comprehensiveness.

### Sampling and procedures

Recruitment targeted adults in the general population who engaged with digital health content and dietary practices—reflected in the predominance of young, highly educated female respondents. Participation was voluntary, and informed consent was obtained electronically prior to enrollment.

### Statistical analysis

Data were analyzed using JMP Pro Version 18 and Microsoft Excel (20, 21). Descriptive statistics were computed to summarize participant characteristics. Associations between categorical variables were assessed using the chi-squared and Fisher’s exact tests, with statistical significance set at a *p*-value of < 0.05 (two-tailed). Missing data were excluded listwise without imputation.

A logistic regression analysis was conducted to examine the association between micronutrient supplementation and diet adherence duration. For this analysis, three dietary categories—vegan, Mediterranean, and gluten-free—were merged into a single “Other Diets” group due to small cell counts that resulted in unstable model estimates. This grouping improved statistical stability and allowed for more reliable odds ratio estimation.

### Ethical considerations

Ethical approval was obtained from the Institutional Review Board of Princess Nourah Bint Abdulrahman University (Approval No. 24-0931). Participation was anonymous, and data confidentiality was maintained throughout the study.

## Results

Table 1 presents sociodemographic and nutrition-related characteristics. The majority of participants were aged 18–24 years (58%) and female (71.5%). A similar proportion (71.5%) held a bachelor’s degree or higher. Approximately half of the participants were overweight or obese (44%), while 43.25% had a normal weight. Approximately one-third (37%) reported following a diet within the previous 6 months. Supplement use was reported by 45.11% of participants. Among supplement users, 63% obtained supplements via prescription, with female participants being significantly more likely than male participants to do so (*χ*^2^(1, *N* = 130) = 19.9, *p* < 0.0001).

**Table 1:** Sociodemographic and nutrition-related characteristics.

Variable	*N*	% of Total
Age		
18–24 years	232	58%
25–35 years	93	23.25%
36–45 years	75	18.75%
Sex		
Female	286	71.50%
Male	114	28.50%
Educational level		
High school or below	86	21.50%
Diploma	28	7%
Bachelor’s degree or more	286	71.50%
BMI		
Underweight	51	12.75%
Normal weight	173	43.25%
Overweight/obese	176	44%
Followed any diet in the past 6 months		
Yes	148	37%
No	252	63%
Consume micronutrients		
Yes	180	45.11%
No	219	54.89%

**Figure 1:**
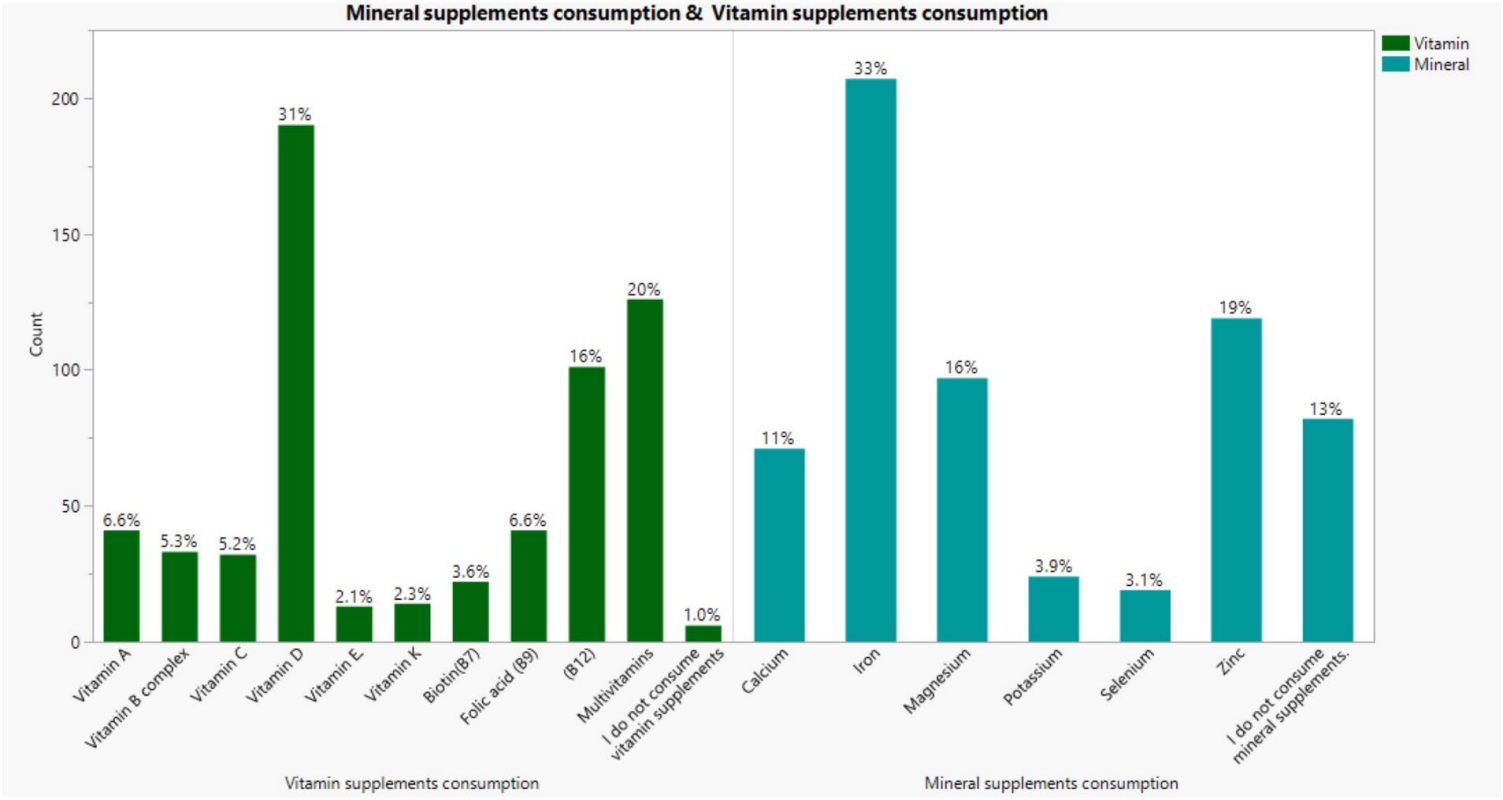
Overview of micronutrient consumption.

Figure 1 shows an overview of micronutrient consumption. Among vitamins, vitamin D was most commonly used (31%), followed by multivitamins (20%) and vitamin B12 (16%). For minerals, iron was the most frequently consumed (33%), followed by zinc (19%) and magnesium (16%).

Table 2 summarizes the association between micronutrient supplement consumption and dietary regimen. Supplement use was most common among individuals following gluten-free (75%) and keto (66.67%) diets. Although supplement users were more likely to follow gluten-free (*p* = 0.0445) and low-carbohydrate (*p* = 0.0456) diets, there was no significant association between supplement consumption and overall dietary regimens.

**Table 2:** Association between micronutrient consumption and dietary regimens.

Diet type	Not consume micronutrients	Consume micronutrients	Total	*P*-value Fisher’s exact test two-tailed
Keto	12 (33.33)	24 (66.67)	36	0.1763
Intermittent fasting	43 (44.33)	54 (55.67)	97	1.0000
Calorie restriction	45 (42.45)	61 (57.55)	106	0.5792
Low carb	28 (35.9)	50 (64.1)	78	**0.04558** ^*^
DASH	7 (35)	13 (65)	20	0.4701
Vegan	9 (34.62)	17 (65.38)	26	0.3844
Mediterranean	10 (34.48)	19 (65.52)	29	0.2984
Gluten-free	6 (25)	18 (75)	24	**0.0445** ^*^
Low fat	22 (35.48)	40 (64.52)	62	0.0924

* A *p*-value of < 0.05 was considered statistically significant.

All values are presented in numbers (percentages) unless indicated otherwise. Bold values and asterisks (*) indicate statistically significant associations between micronutrient consumption and dietary patterns.

**Figure 2:**
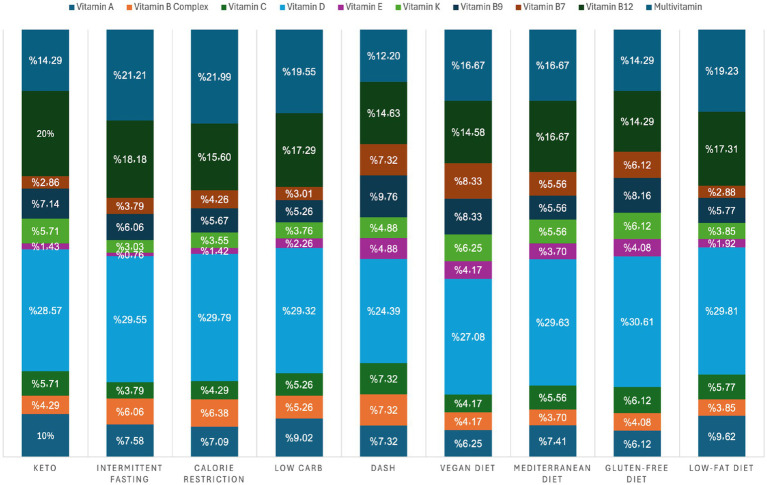
Diet type and vitamin consumption.

Figure 2 shows that vitamin D was the most commonly consumed supplement across all dietary patterns, with the highest use among individuals following gluten-free (30.61%), low-fat (29.81%), and calorie-restricted diets (29.79%). Several diets demonstrated significant associations with specific vitamins: keto and low-carb diets were associated with multiple vitamins (A, D, K, and B12), and a low-carb diet was also associated with vitamin C and multivitamins. The DASH, vegan, Mediterranean, gluten-free, and low-fat diets also showed selective associations, particularly with vitamins E, K, and folate. In contrast, intermittent fasting and calorie-restricted diets showed similar intake trends but no significant vitamin-diet associations. Overall, vitamin D was the most commonly used supplement across diet types, with certain diets showing unique associations with certain vitamins.

**Figure 3:**
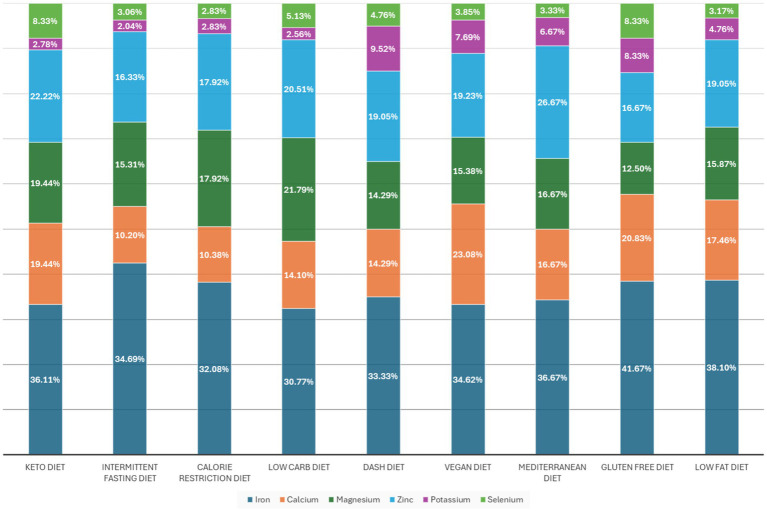
Diet type and mineral consumption.

Figure 3 illustrates mineral consumption across diet types, showing that iron is the most frequently consumed mineral in all groups, with the highest intake among individuals following gluten-free (41.7%), low-fat (38.1%), and Mediterranean (36.6%) diets. Zinc and magnesium were the next most common minerals, particularly among those following keto, intermittent fasting, or calorie-restricted diets. Several diets showed notable mineral-diet associations, including keto (calcium, *p* = 0.0186; selenium, *p* = 0.0166), low-carb (calcium, *p* = 0.0416), DASH (potassium, *p* = 0.0085), vegan (calcium, *p* = 0.0090; potassium, *p* = 0.0240), Mediterranean (potassium, *p* = 0.0434), gluten-free (calcium, *p* = 0.0375; potassium, *p* = 0.0166), and low-fat (calcium, *p* = 0.0042; potassium, *p* = 0.0421). Overall, iron dominates mineral supplementation, while calcium and potassium demonstrate significant associations with multiple dietary patterns.

Table 3 presents the distribution of popular diet types by sex and BMI. The calorie-restricted diet was the most widely adopted (71.62%) and maintained for the longest duration (29.05% for 4–6 months). Intermittent fasting was most popular among female participants (56.08%), while only 10.14% of male participants followed it, indicating a significant sex difference (*p* = 0.0011). Male participants most commonly chose the calorie-restricted diet (18.24%). Calorie restriction was also the most commonly followed diet among both normal-weight (31.08%) and overweight/obese participants (37.84%), with a significant association with BMI (*p* = 0.0441). Among underweight participants, calorie restriction, intermittent fasting, and low-carb diets were followed equally (2.70%). The low-carb diet showed significant associations with age (*p* = 0.0484; more common among 18–24-year-olds) and sex (*p* = 0.0344; more common among female participants). The gluten-free diet was significantly more commonly followed by individuals aged 36–45 (*p* = 0.0191).

**Table 3:** Distribution of popular diet types by sex and BMI.

Diet type	Sex		BMI			Overall
	Female	Male	Underweight	Normal weight	Overweight/Obese	
Keto	31 (20.95)	5 (3.38)	2 (1.35)	8 (5.41)	26 (17.57)	36 (24.32)
Intermittent fasting	83 (56.08)	15 (10.14)	4 (2.70)	39 (26.35)	55 (37.16)	98 (66.22)
Calorie restriction	79 (53.38)	27 (18.24)	4 (2.70)	46 (31.08)	56 (37.84)	106 (71.62)
Low carb	65 (43.92)	13 (8.78)	4 (2.70)	33 (22.30)	41 (27.70)	78 (52.70)
DASH	16 (10.81)	5 (3.38)	2 (1.35)	6 (4.05)	13 (8.78)	21 (14.19)
Vegan	22 (14.86)	4 (2.70)	2 (1.35)	10 (6.76)	14 (9.46)	26 (17.57)
Mediterranean	26 (17.57)	4 (2.70)	3 (2.03)	11 (7.43)	16 (10.81)	30 (20.27)
Gluten-free	21 (14.19)	3 (2.03)	2 (1.35)	6 (4.05)	16 (10.81)	24 (16.22)
Low fat	49 (33.11)	14 (9.46)	2 (1.35)	28 (18.92)	33 (22.30)	63 (42.57)

Percentages are based on the total number of diet followers in the past 6 months (*N* = 148). Some participants may follow more than one diet.

Table 4 presents the association between micronutrient consumption and the duration of adherence to different dietary regimens. No significant associations were observed for the majority of diets; however, participants practicing intermittent fasting for 4–6 months had significantly higher odds of consuming supplements (OR = 3.96, *p* = 0.012). Similarly, individuals adhering to the combined ‘Other Diets’ group for 4–6 months showed markedly increased supplement use (OR = 7.70, *p* = 0.008).

**Table 4:** Association between micronutrient consumption and diet adherence duration across dietary regimens.

Diet adherence duration (months)	Consume micronutrients	Not consume micronutrients	OR	95% CI	*P*-value
Keto					0.526
>1	12 (50)	8 (66.6)	1	–	–
1–3	7 (29.2)	3 (25)	1.555	0.320–8.916	0.589
4–6	5 (20.8)	1 (8.3)	3.333	0.425–70.287	0.270
Intermittent fasting					**0.042** ^*^
>1	13 (24)	18 (41.8)	1	–	–
1–3	21 (38.9)	18 (41.8)	1.615	0.627–4.245	0.321
4–6	20 (37)	7 (16.2)	3.956	1.335–12.734	**0.012** ^*^
Calorie restriction					0.365
>1	11 (18)	12 (26.6)	1	–	–
1–3	22 (36)	18 (40)	1.333	0.476–3.774	0.583
4–6	28 (45.9)	15 (33.3)	2.036	0.728–5.808	0.174
Low carb					0.754
>1	19 (38)	12 (44.4)	1	–	–
1–3	19 (38)	8 (29.6)	1.5	0.504–4.622	0.466
4–6	12 (24)	9 (25.9)	1.082	0.334–3.625	0.894
Low fat					0.532
>1	11 (27.5)	9 (40.9)	1	–	–
1–3	14 (35)	7 (31.8)	1.636	0.464–5.970	0.443
4–6	15 (37.5)	6 (27.2)	2.045	0.569–7.785	0.273
Other diets					**0.020** ^*^
>1	10 (31)	11 (73)	1	–	–
1–3	8 (25)	2 (13)	4.4	0.851–34.033	0.078
4–6	14 (44)	2 (13)	7.7	1.617–57.307	**0.008** ^*^

All values are presented in numbers (percentages) unless indicated otherwise. Bold values and asterisks (*) indicate statistically significant associations between micronutrient consumption and dietary patterns.

## Discussion

In Saudi Arabia, the growing popularity of various dietary trends among young adults highlights the need to understand their nutritional effects. This study examines the link between dietary regimens and micronutrient supplementation, as different diets can influence both the amount and type of vitamins consumed, highlighting the relevance of this research.

### Dietary regimens

Our findings reported that approximately one-third of participants had followed at least one structured diet in the previous 6 months. The most commonly adopted diet was calorie restriction, a pattern consistent with its reputation for simplicity and flexibility. Unlike diets that eliminate entire food groups, calorie restriction can be practiced without significant lifestyle disruption, likely contributing to its popularity. This contrasted with previous findings, which reported much lower engagement with calorie-restricted diets in a Saudi sample (22). Approximately half of the participants reported practicing intermittent fasting, which mirrors previous findings in Riyadh, where a high prevalence of intermittent fasting was also observed (23). Its appeal may stem from cultural familiarity with fasting and its perceived effectiveness.

Interestingly, our study found that women were significantly more likely than men to adopt intermittent fasting. This pattern aligns with findings that identified gender as a key factor influencing adoption (24). This could reflect the greater social and psychological pressure on women to conform to certain body ideals and gendered differences in health behaviors. Low-carbohydrate diets were frequently reported by participants in this study, with adoption levels noticeably higher than those documented in earlier Saudi research (4) and European contexts such as Sweden (25). This rise in popularity may reflect increased public interest in reducing carbohydrate intake as a strategy for weight control and improved metabolic health. The influence of social media, fitness trends, and widespread discussions around the effects of carbs may have contributed to the growing appeal of this diet, particularly among younger and working-age individuals, who comprised the majority of our sample. A moderate portion of the participants adopted low-fat, Mediterranean, and DASH diets. Compared to older findings, adoption of low-fat diets appears to be more common in our sample than in some previous studies but lower than among specific populations such as medical students (4, 26). These variations may be due to differences in nutritional literacy or access to professional dietary guidance. A good portion of participants in our study followed the Mediterranean diet, showing higher adherence compared to earlier Saudi research (27), which reported a much lower rate. This may reflect growing awareness of the diet’s health benefits and increased exposure through the media and healthcare professionals.

In contrast, although several participants reported following the DASH diet, findings from the UAE suggest that overall adherence and awareness of this diet remain low in the region (28). This may indicate that, unlike the Mediterranean diet, the DASH approach has not yet gained significant traction among the public. Vegan diets were reported by approximately one-third of participants, substantially higher than those documented in previous Saudi studies (22). This shift may reflect growing interest in plant-based lifestyles, possibly influenced by ethical considerations, health claims, or social trends. A quarter of participants also practiced gluten-free diets, much lower than earlier reports of short-term experimentation (4). The discrepancy may reflect differences in how the diet is defined or practiced, whether adopted for medical necessity, weight management, or perceived health benefits.

The popularity of specific dietary regimens in Saudi Arabia is evident in the studied population. Intermittent fasting is widely practiced among Saudis, both during and outside of Ramadan (24). In comparison, a minority of participants followed ketogenic and gluten-free diets, with adherence generally short-term (4). These findings may reflect differences in familiarity, accessibility, and adherence to each dietary regimen within this population.

### Micronutrient consumption

Approximately half of the participants reported using supplements, supporting existing evidence that supplement consumption is widespread in Saudi Arabia. These findings are comparable to previous national studies that have documented similarly high usage rates, reinforcing the growing reliance on supplementation as part of modern health practices (11, 29). Among specific vitamins, vitamin D emerged as the most commonly used, followed by multivitamins and Vitamin B12. This pattern mirrors findings from earlier studies in Saudi Arabia and neighboring Jordan (30, 31). Vitamin D deficiency remains a common issue due to limited sun exposure and lifestyle habits that reduce natural vitamin synthesis. Females were significantly more likely than males to use micronutrient supplements, mostly by prescription. Alfawaz et al. similarly found higher supplement use among Saudi female adolescents (33% vs. 17.9%) (32). Both results confirm that sex strongly influences supplement consumption in Saudi populations.

The widespread use of vitamin D reflects public awareness of this deficiency and may increase screening and prescribing efforts by healthcare professionals. The majority of participants reported using prescribed supplements when examining supplementation sources, while a smaller portion relied on over-the-counter or self-prescribed options. This trend is consistent with local studies that noted healthcare providers’ strong role in supplement distribution, especially pharmacists (29). However, it contrasts with findings from Jordan, where self-prescription was more common (31), indicating that cultural and healthcare system differences may shape supplement access and trust in professional guidance. Beyond general consumption patterns, this study also assessed the association between dietary regimens and supplement use. The majority of individuals following gluten-free and low-carb diets reported using supplements, which aligns with international research emphasizing the risk of micronutrient deficiencies on restrictive diets. Gluten-free diets have been associated with inadequate intake of iron, folate, vitamin D, and B12, while low-carb regimens may reduce essential nutrients such as thiamine, calcium, and magnesium (33, 34). Supplement use among these groups may reflect a preventive strategy and a response to emerging deficiency symptoms. Across nearly all dietary groups, vitamin D remained the most commonly used supplement, a trend consistent with international literature on restricted diets such as low-calorie (35), vegan (36), and gluten-free plans (37). Participants following ketogenic and low-carb diets also showed frequent use of vitamin D, multivitamins, and B12, further supporting evidence that these diets, although popular, may create nutritional gaps that require careful management (17).

Moreover, our findings on the significant associations between specific dietary regimens and mineral intake are in line with existing international literature. Among individuals following vegetarian or vegan diets, iron intake emerged as a major concern. A meta-analysis of 24 cross-sectional studies confirmed that adult vegetarians tend to have significantly lower serum ferritin levels compared to non-vegetarians (17). Similarly, vegans have been found to consistently consume less calcium than both vegetarians and omnivores (*p* < 0.0001), highlighting the importance of ongoing calcium monitoring within this population (38). Participants following low-carbohydrate or ketogenic diets have also demonstrated patterns of potential micronutrient insufficiency. Evidence from clinical trials and systematic reviews has shown that these diets are commonly associated with reduced intake or levels of calcium, magnesium, iron, and thiamine, with some studies even reporting declines in serum calcium over time (19, 39).

The lower calcium status observed in ketogenic dieters may result from limited consumption of dairy and legumes, as well as potential changes in bone metabolism due to chronic metabolic acidosis, which can increase urinary calcium loss (40). While initial serum values may remain within normal ranges, prolonged adherence to such diets without adequate planning poses risks that may necessitate supplementation.

Gluten-free diets, although essential for individuals with celiac disease, may also negatively affect mineral absorption and bone health. A long-term study revealed that despite higher calcium intake, individuals on gluten-free diets had lower fractional calcium absorption and significantly reduced bone mineral density (BMD) compared to controls (*p* < 0.001 and *p* < 0.05, respectively) (41). These findings suggest that increased intake alone may not fully compensate for impaired calcium absorption, emphasizing the need for ongoing bone health monitoring in this group.

Additionally, low-fat diets—which are often perceived as healthy and balanced—may indirectly impair calcium metabolism. This occurs through reduced absorption of fat-soluble vitamin D, a nutrient critical for calcium uptake. Clinical research shows that consuming vitamin D with low-fat meals leads to significantly lower serum 25(OH)D levels, potentially decreasing calcium absorption efficiency (42). This underscores the frequently overlooked risk of micronutrient disruption, even in well-regarded dietary patterns. Taken together, these findings underscore a broader understanding: restrictive dietary patterns—whether motivated by medical needs, ethical beliefs, or weight management goals—can inherently increase the risk of mineral imbalances. The supplementation behaviors observed among participants in this study likely reflect both professional dietary guidance and individual efforts to manage or prevent such deficiencies.

### Strengths and limitations

This study offers an important contribution as one of the first investigations in Riyadh to examine micronutrient supplement use in the context of popular dietary regimens, supported by an adequate sample size and coverage of multiple diet types. However, the reliance on non-probability convenience sampling through online platforms may have introduced selection bias, particularly given the high proportion of young, highly educated female participants, which limits the generalizability of findings to the wider Saudi adult population. Additionally, the use of self-reported data for supplement intake and diet adherence may be influenced by recall or social desirability bias. The study also did not assess actual dietary nutrient intake, which limits the ability to determine micronutrient sufficiency among different diet groups. Moreover, some diet categories were merged due to small sample sizes, which may limit the ability to detect more nuanced differences between individual restrictive diets.

## Conclusion

This study provides valuable insight into the relationship between dietary regimens and micronutrient supplementation among adults in Riyadh. The findings reveal that approximately half of the participants used vitamin and mineral supplements, with vitamin D being the most commonly consumed vitamin across all diet types. Similarly, iron emerged as the most frequently consumed mineral, particularly among followers of gluten-free, ketogenic, and Mediterranean diets. Notably, individuals adhering to gluten-free, ketogenic, and low-carbohydrate diets reported higher rates of supplement use, reflecting their increased risk of nutritional deficiencies. Statistically significant associations were observed between certain diets and specific vitamins and minerals, reinforcing international evidence that restrictive eating patterns may compromise nutritional status. These results underscore the importance of professional dietary guidance when adopting specialized diets and highlight the need for public health interventions that promote balanced nutrition and responsible supplement use. Moreover, longer adherence to certain restrictive diets, particularly intermittent fasting and combined plant-based/gluten-free patterns, was associated with higher supplement use.

## Data Availability

The original contributions presented in the study are included in the article/Supplementary material, further inquiries can be directed to the corresponding author.

## References

[1] Lombardo CA. The role of diets as triggering factors for binge eating disorder in patients with overweight/obesity. Master thesis, University of Padova. (2023–2024). Available online at: https://thesis.unipd.it/retrieve/e6edd860-b378-4e9f-b552-33ee177cc0a9/Elaborato%20finale%20Lombardo%20Carlotta%20Anastasia.pdf (Accessed May 7, 2025).

[2] Stice E Rohde P Gau J Shaw H. An effectiveness trial of a dissonance-based eating disorder prevention program for high-risk adolescent girls. J Consult Clin Psychol. (2009) 77:825–34. doi: 10.1037/a0016132, 19803563 PMC2760014

[3] van Hoeken D Hoek HW. Review of the burden of eating disorders: mortality, disability, costs, quality of life, and family burden. Curr Opin Psychiatry. (2020) 33:521–7. doi: 10.1097/YCO.0000000000000641, 32796186 PMC7575017

[4] Alhusseini N Alsinan N Almutahhar S Khader M Tamimi R Elsarrag MI . Dietary trends and obesity in Saudi Arabia. Front Public Health. (2024) 11:11. doi: 10.3389/fpubh.2023.1326418 PMC10808649 38274536

[5] Agras W Stewart R Roninson A. The Oxford handbook of eating disorders. Oxford: Oxford University Press (2017).

[6] Dowis K Banga S. The potential health benefits of the ketogenic diet: a narrative review. Nutrients. (2021) 13:1654. doi: 10.3390/nu13051654, 34068325 PMC8153354

[7] Healthdirect Australia. Ketogenic diet. (2024). Available online at: https://www.healthdirect.gov.au/ketogenic-diet (Accessed May 7, 2025).

[8] Johns Hopkins Medicine. Intermittent fasting: what is it, and how does it work? (2024). Available online at: https://www.hopkinsmedicine.org/health/wellness-and-prevention/intermittent-fasting-what-is-it-and-how-does-it-work (Accessed May 7, 2025).

[9] Memorial Sloan Kettering Cancer Center. Low-calorie diet. (2025). Available online at: https://www.mskcc.org/experience/patient-support/nutrition-cancer/diet-plans-cancer/low-calorie-diet (Accessed May 7, 2025).

[10] World Health Organization. Micronutrients. (2019). Available online at: https://www.who.int/health-topics/micronutrients#tab=tab_1 (Accessed May 7, 2025).

[11] Algaeed HA AlJaber MI Alwehaibi AI AlJaber LI Arafah AM Aloyayri MA . General public knowledge and use of dietary supplements in Riyadh, Saudi Arabia. J Family Med Prim Care. (2019) 8:3147–54. doi: 10.4103/jfmpc.jfmpc_511_19, 31742134 PMC6857379

[12] Alhashem AM Alghamdi RA Alamri RS Alzhrani WS Alrakaf MS Alzaid NA . Prevalence, patterns, and attitude regarding dietary supplement use in Saudi Arabia: data from 2019. PLoS One. (2022) 17:e0274412. doi: 10.1371/journal.pone.0274412, 36129901 PMC9491604

[13] U.S. Food and Drug Administration. Understanding Dietary Supplements. (2022). [Report/Brief]. [If available add URL] (Accessed May 7, 2025).

[14] Martiniaková M Babíková M Mondocková V Blahová J Kováčová V Omelka R. The role of macronutrients, micronutrients and flavonoid polyphenols in the prevention and treatment of osteoporosis. Nutrients. (2022) 14:523. doi: 10.3390/nu14030523 35276879 PMC8839902

[15] Sakkas H Bozidis P Touzios C Kolios D Athanasiou G Athanasopoulou E . Nutritional status and the influence of the vegan diet on the gut microbiota and human health. Medicina Kaunas. (2020) 56:88. doi: 10.3390/medicina56020088, 32098430 PMC7073751

[16] Niklewicz A Smith AD Smith A Holzer A Klein A McCaddon A . The importance of vitamin B12 for individuals choosing plant-based diets. Eur J Nutr. (2023) 62:1551–9. doi: 10.1007/s00394-022-03025-4, 36469110 PMC10030528

[17] Haider LM Schwingshackl L Hoffmann G Ekmekcioglu C. The effect of vegetarian diets on iron status in adults: a systematic review and meta-analysis. Crit Rev Food Sci Nutr. (2018) 58:1359–74. doi: 10.1080/10408398.2016.1259210, 27880062

[18] Detopoulou P Papadopoulou SK Voulgaridou G Dedes V Tsoumana D Gioxari A . Ketogenic diet and vitamin D metabolism: a review of evidence. Meta. (2022) 12:1288. doi: 10.3390/metabo12121288, 36557329 PMC9788458

[19] Kenig S Petelin A Vatovec TP Mohorko N Jenko-Pražnikar Z. Assessment of micronutrients in a 12-week ketogenic diet in obese adults. Nutrition. (2019) 67–68:110522. doi: 10.1016/j.nut.2019.06.003 31445313

[20] JMP Statistical Discovery. JMP® statistical discovery (SAS). (2025). Available online at: https://www.jmp.com (Accessed May 7, 2025).

[21] Microsoft. Excel | Microsoft 365. (2025). Available online at: https://www.microsoft.com/microsoft-365/excel. (Accessed May 7, 2025).

[22] AlHusseini N Sajid M Akkielah YA Khalil T Alatout M Cahusac P . Vegan, vegetarian and meat-based diets in Saudi Arabia. Cureus. (2021) 13:e18073. doi: 10.7759/cureus.18073, 34692294 PMC8523473

[23] Alsaleh SS Al Mana AKA Saleh SM Ali SA Samreen S Ali WS. Intermittent fasting among individuals in Saudi Arabia: a cross-sectional analysis of characteristics, reasons, and health outcomes [preprint]. (2024). Available online at: https://www.researchgate.net/publication/387435624_Intermittent_Fasting_among_Individuals_in_Saudi_Arabia_A_Cross-sectional_Analysis_of_Characteristics_Reasons_and_Health_Outcomes (Accessed May 7, 2025).

[24] Alnasser A Almutairi M. Considering intermittent fasting among Saudis: insights into practices. BMC Public Health. (2022) 22:592. doi: 10.1186/s12889-022-12908-4, 35346130 PMC8959076

[25] Bärebring L Winkvist A Augustin H. Sociodemographic factors associated with reported attempts at weight loss and specific dietary regimens in Sweden: the SWEDIET-2017 study. PLoS One. (2018) 13:e0197099. doi: 10.1371/journal.pone.0197099, 29746536 PMC5944954

[26] Alissa EM Alsawadi H Zedan A Alqarni D Bakry M Hli NB. Knowledge, attitude and practice of dietary and lifestyle habits among medical students in king Abdulaziz university, Saudi Arabia. Int J Food Sci Nutr. (2015) 4:650–5. doi: 10.11648/j.ijnfs.20150406.18

[27] Alnabulsi M Imam AA Alawlaqi AA Alhawaj FH Jamjoom GF Alsaeidi LD . Adherence to the Mediterranean diet in Saudi Arabia and its association with socioeconomic status and depression. Medicina (Kaunas). (2024) 60:642. doi: 10.3390/medicina60040642, 38674290 PMC11051785

[28] Hashim M Radwan H Ismail LC Faris ME Mohamad MN Saleh ST . Determinants for Mediterranean diet adherence beyond the boundaries: a cross-sectional study from Sharjah, the United Arab Emirates. J Transl Med. (2024) 22:513. doi: 10.1186/s12967-024-05172-0, 38807139 PMC11134895

[29] Alshehri AA Alqahtani S Aldajani R Alsharabi B Alzahrani W Alguthami G . Knowledge, attitudes, and practices of dietary supplement use in Western Saudi Arabia: a cross-sectional study. Nutrients. (2025) 17:1233. doi: 10.3390/nu17071233, 40218991 PMC11990556

[30] Abdel-Salam DM Alruwaili JM Alshalan RA Alruwaili TA Alanazi SA Lotfy AMM. Epidemiological aspects of dietary supplement use among Saudi medical students: a cross-sectional study. Open Public Health Open Public Health. (2020) 13:783. doi: 10.2174/1874944502013010783

[31] Basheer HA Elsalem L Jaber D Ibraheem SM Alhamad H Jum'ah AA. Knowledge, awareness and practices regarding dietary supplements in Jordan. Trop J Pharm Res. (2021) 20:649–59. doi: 10.4314/tjpr.v20i3.30

[32] Alfawaz HA Khan N Aljumah AA Hussain SD Khattak MN Al-Daghri NM. The association between vitamin D status and supplementation among Saudi female adolescents. Int J Environ Res Public Health. (2020) 17:351532443434

[33] Kreutz JM Heynen L Vreugdenhil ACE. Nutrient deficiencies in children with celiac disease during long-term follow-up. Clin Nutr. (2023) 42:1175–80. doi: 10.1016/j.clnu.2023.05.003, 37246082

[34] Churuangsuk C Catchpole A Talwar D Welsh P Sattar N Lean MEJ . Low thiamine status in adults following low-carbohydrate/ketogenic diets: a cross-sectional comparative study of micronutrient intake and status. Eur J Nutr. (2024) 63:2667–79. doi: 10.1007/s00394-024-03459-y, 38967675 PMC11490449

[35] Damms-Machado A Weser G Bischoff SC. Micronutrient deficiency in obese subjects undergoing low calorie diet. Nutr J. (2012) 11:34. doi: 10.1186/1475-2891-11-34 22657586 PMC3404899

[36] Bakaloudi DR Halloran A Rippin HL Oikonomidou AC Dardavesis TI Williams J . Intake and adequacy of the vegan diet: a systematic review of the evidence. Clin Nutr. (2021) 40:3503–21. doi: 10.1016/j.clnu.2020.11.035, 33341313

[37] Alhosain AI Alshammari GM Almoteri BL Mohammed MA Binobead MA Yahya MA. Long-term effect of gluten-free diets on nutritional status, body composition, and associated factors in adult Saudi females with celiac disease. Nutrients. (2022) 14:2090. doi: 10.3390/nu14102090 35631231 PMC9144408

[38] Bickelmann FV Leitzmann MF Keller M Baurecht H Jochem C. Calcium intake in vegan and vegetarian diets: a systematic review and meta-analysis. Crit Rev Food Sci Nutr. (2023) 63:10659–77. doi: 10.1080/10408398.2022.2084027, 38054787

[39] Churuangsuk C Griffiths D Lean MEJ Combet E. Impacts of carbohydrate-restricted diets on micronutrient intakes and status: a systematic review. Obes Rev. (2019) 20:1132–47. doi: 10.1111/obr.12857, 31006978

[40] Garofalo V Barbagallo F Cannarella R Calogero AE La Vignera S Condorelli RA. Effects of the ketogenic diet on bone health: a systematic review. Front Endocrinol (Lausanne). (2023) 14:1042744. doi: 10.3389/fendo.2023.1042744 36817595 PMC9932495

[41] Pazianas M Butcher GP Subhani JM Finch PJ Ang L Collins C . Calcium absorption and bone mineral density in celiacs after long-term treatment with gluten-free diet and adequate calcium intake. Osteoporos Int. (2005) 16:56–63. doi: 10.1007/s00198-004-1641-2, 15221205

[42] Raimundo FV Lang MAB Scopel L Marcondes NA Araújo MGA Faulhaber GAM . Effect of fat on serum 25-hydroxyvitamin D levels after a single oral dose of vitamin D in young healthy adults: a double-blind randomized placebo-controlled study. Eur J Nutr. (2015) 54:391–6. doi: 10.1007/s00394-014-0718-8 24853643

