# Correction: Micronutrient supplementation among adults following restrictive diets in Riyadh: a cross-sectional study

**DOI:** 10.3389/fnut.2026.1812642

**Published:** 2026-03-04

**Authors:** Rehab Aldahash, Haifa Alturki, Joury Dabbour, Shahad Aldossari, Shahad Alonazi, Sarah Aldhfayan

**Affiliations:** Department of Health Sciences, College of Health and Rehabilitation Sciences, Princess Nourah Bint Abdulrahman University, Riyadh, Saudi Arabia

**Keywords:** dietary regimens, iron, micronutrients, Saudi Arabia, supplementation, vitamin D

In the published article, there was a mistake in the **Funding** Statement. The grant number provided for Princess Nourah bint Abdulrahman University Researchers Supporting Project was displayed as “PNURSP2025R533.” The correct number is “PNURSP2026R533.”

Funding

The author(s) declared that financial support was received for this work and/or its publication. This research was funded by Princess Nourah bint Abdulrahman University Researchers Supporting Project number (PNURSP2026R533), Princess Nourah bint Abdulrahman University, Riyadh, Saudi Arabia.

The original version of this article has been updated.

