# Supplementary material for: Micronutrient supplementation among adults following restrictive diets in Riyadh: a cross-sectional study
Source: Front Nutr. 2026 Feb 12;13:1686609. doi: 10.3389/fnut.2026.1686609 (PMC12935628; doi:10.3389/fnut.2026.1686609)
Supplement: Supplementary file 1 [file Table_1.pdf]

Questionnaire

1. Sociodemographic characteristics

1. How old are you?

- 18-24 years
- 25-35 years
- 36-45 years

2. What is your gender?

- Male
- Female

3. What is your educational Level?

- Less than high school
- High school education
- Bachelor’s degree
- Postgraduate studies

4. What is your height in centimetres? ______

5. What is your weight in kilograms? _____

2. Diet tool

1) Which diet did you follow in the past 6 months?

	Duration of diet			
	Less than 1 month	1-3 months	4-6 months	I did not follow this diet
Keto				
Intermittent fasting				
Calorie restriction				
Low-carb diet				
DASH diet				
Vegan				
Mediterranean diet				
Gluten-free diet				
Low-fat diet				
Other (mention)				

2) How did you learn about the diet/s?

- Healthcare professional (e.g., doctor, dietitian, health educator)
- Family member or friend
- Online sources (websites, blogs, social media)
- Books or printed publications

3) What challenges have you faced while following the diet/s? (Select all that apply)

- Financial challenges (e.g., Cost of healthy food options)
- Difficulty in adhering
- Lack of support from family or friends
- Health issues
- I did not face any challenges

4) How would you evaluate your progress with the diet plan?

- Successfully achieved the goal
- On the way to the goal
- Stopped without achieving the goal

5) Do you consume vitamins/minerals?

- Yes
- No

If yes

1- Which of the following vitamin supplements do you consume? (Select all that apply)

- Vitamin A
- Vitamin B complex (B1, B2, B3, B6, B12, etc.)
- Vitamin C
- Vitamin D
- Vitamin E
- Vitamin K
- Folic acid (B9)
- Biotin(B7)
- Multivitamins
- I do not consume vitamin supplements

2- Which of the following mineral supplements do you consume? (Select all that apply)

- Calcium
- Magnesium
- Iron
- Zinc
- Potassium
- Selenium
- I do not consume mineral supplements.

3- What is the source you relied on for consuming vitamin/mineral?

- Prescription
- Without prescription

4- What is the duration of your vitamin/mineral consumption?

- Less than a month
- 1-3 months
- 4-6 months

Eating disorders:

- If you have been diagnosed with an eating disorder, what type were you diagnosed with?

- Anorexia nervosa
- Bulimia nervosa
- Binge eating disorder
- Other (please specify): ___
- I have not been diagnosed with any eating disorder.

If you haven’t been diagnosed

EAT-26 Test

Instructions: Please read each statement carefully and indicate how much you agree with it based on your personal experience over the past three months.

1. I am terrified about being overweight.
2. I avoid eating when I am hungry.
3. I find myself preoccupied with food.
4. I have gone on eating binges where I feel that I may not be able to stop.
5. I cut my food into small pieces.
6. I’m aware of the calorie content of foods that I eat.
7. I particularly avoid food with a high carbohydrate content (i.e. bread, rice, potatoes, etc.)

8. I feel that others would prefer if I ate more.

9. I vomit after I have eaten

10. I feel extremely guilty after eating.

11. I am occupied with a desire to be thinner.

12. I think about burning up calories when I exercise.

13. Other people think that I am too thin.

14. I am preoccupied with the thought of having fat on my body.

15. I take longer than others to eat my meals.

16. I avoid foods with sugar in them.

17. I eat diet foods.

18. I feel that food controls my life.

19. I display self-control around food.

20. I feel that others pressure me to eat.

21. I give too much time and thought to food.

22. I feel uncomfortable after eating sweets.

23. I engage in dieting behavior.

24. I like my stomach to be empty.

25. I have the impulse to vomit after meals.

26. I enjoy trying new rich foods.

- Always
- Usually
- Often
- Sometimes
- Rarely
- Never

This is the end of the questions

Thank you for participating
